# Doctors’ experience providing primary care for refugee women living with chronic pain: a qualitative study

**DOI:** 10.1186/s12913-024-11506-x

**Published:** 2024-09-27

**Authors:** Areni Altun, Helen Brown, Elizabeth Sturgiss, Grant Russell

**Affiliations:** 1 Eastern Health Clinical School, Monash University, Melbourne, VIC Australia; 2 Deakin University, Melbourne, Australia; 3 Monash University, Melbourne, Australia

**Keywords:** Primary care, Chronic pain, Qualitative, Refugee women, Care experience

## Abstract

**Background:**

The experiences of GPs in Australia highlight key considerations regarding workload demands, remuneration incentives and the practical implications of working in regions with high ethnic density. This exploration helps to understand the elements that influence GPs delivery of care, particular for refugee women who exhibit disproportionately higher rates of chronic pain. This qualitative study explored the experiences of GPs providing care for refugee women living with chronic pain.

**Methods:**

Semi-structured interviews were undertaken with 10 GPs (9 female and 1 male) practicing across metropolitan Melbourne, Australia. GPs were recruited via purposive sampling and a snowballing strategy. Participants work experience ranged from one to 32 years. Audio recordings of the interviews were transcribed verbatim and stored in qualitative data Nvivo 12 software for coding. Transcripts of interviews were analysed thematically using a phenomenological approach.

**Results:**

Three overarching themes were identified: (1) meeting the needs of refugee women living with chronic pain; (2) the role of the GP; and (3) the challenges of the health care system. These themes reflected the complexity of consultations which arose, in part, from factors such as trust, the competencies of clinician’s and the limitations posed by time, funding and interpreter use.

**Conclusion:**

GPs acknowledged the uniqueness of refugee women’s chronic pain needs and whilst doctors welcomed care, many were often challenged by the complex nature of consultations. Those that worked in settings that aligned with refugee women’s needs highlighted the importance of cultivating culturally safe clinical environments and listening to their patients’ stories. However, system level challenges such as time, funding and resource constraints created significant challenges for GPs. Exploring GPs experiences allows for a better understanding of how vectors of disadvantage intersect in health care and highlights the need to better support doctors to improve health care provision for refugee women living with chronic pain.

**Supplementary Information:**

The online version contains supplementary material available at 10.1186/s12913-024-11506-x.

## Background

Currently, there are more than 110 million people forcibly displaced worldwide due to persecution, conflict, violence and human rights violations [1], with Australia serving as a primary site for the resettlement of displaced individuals. The Australian Humanitarian Settlement Program adjusts the size and focus of the program annually to respond to evolving humanitarian situations and global resettlement needs. Between 2018 and 2022, the Humanitarian Settlement Program allocated 18,750 positions for displaced persons to resettle [2, 3]. In 2024, this figure increased to 20,000 [2, 3], indicating a return to pre-pandemic refugee population figures [4]. People from refugee backgrounds arriving to Australia face numerous challenges related to their mental and physical well-being, often exhibiting poorer health outcomes compared to the general population [5–7].

Refugee women, in particular, are at a heightened risk of experiencing chronic pain [8]. Disparities in health outcomes persist between people with and without a migration background due to a confluence of factors. These factors, originating from both migration-related experiences and non-immigration-related factors contribute to this health gap. Refugee women are particularly susceptible to encountering various mental and physical health risk factors, including exposure to violence, feelings of insecurity, and challenges related to gender-based abuse. Moreover, in comparison to non-minority compatriots, refugee women experience barriers to accessing healthcare in host countries as a result of language, limited understanding of the healthcare system, and insufficient cultural competence among healthcare providers [9].

Pain is a global health priority and its effective management frequently falls under the purview of general practice. Chronic pain, defined as *pain that lasts longer than three months*, or in many instances *beyond its normal tissue healing time frame*, presents as a major public health challenge for refugee populations and health care providers [10]. In multicultural societies such as Australia, General practitioners (GPs) are increasingly required to provide care to patients from various backgrounds, including those arriving on humanitarian grounds [11, 12]. Women from refugee backgrounds face multiple stressors related to displacement, culture and language, and evidence highlights a number of challenges associated with navigating health care in host countries such as Australia [8, 13, 14]. Refugee women who present to primary care settings often have multiple distressing experiences, including trauma [15], and therefore mental, physical and social health needs are likely to be high and complex [16–19].

In Australia, individuals holding a refugee visa are entitled to the same healthcare rights as Australian citizens. This parity doesn’t extend to asylum seekers. Nonetheless, at the state level, various programs exist to facilitate access to healthcare for asylum seekers. The main burden of addressing refugee and asylum seeker health needs falls to primary care providers. However, people from refugee backgrounds struggle to access care, and primary care services often struggle to provide appropriate care for complex presentations such as chronic pain [20]. According to the Australian Bureau of Statistics, GPs remain the most frequently consulted health professional with 82.3% of the population reported to visit a GP as of 2023 [21]. GPs are often the first point of contact for patients seeking medical care and are integral in the management and coordination of ongoing care for patients with chronic conditions such as pain. However, the local healthcare system of a country shapes the design and implementation of services, and funding within primary care will determine the services available for refugee populations. In Australia and Canada, primary care funding is predominantly based on a fee-for-service model, where GPs are compensated based on individual consultations [22]. In contrast, the United Kingdom (UK) follows a patient enrolment system, where GPs are remunerated through contractual agreements with the National Health Service [22].

Medicare, which is Australia’s nationally funded health care scheme, subsidises the costs of some medical and allied health services [23, 24]. One way Medicare assists GPs is through Chronic Disease Management Plans (CDM) plans which in turn help patients with chronic medical conditions, such as pain, by providing an organised approach to care. A CDM offers a plan of action by the GP that identifies the patients’ health and care needs; sets out the services to be provided by the GP; and lists the actions to take to help manage patient’s chronic condition [25]. Evidence shows that CDM plans are appropriately targeting those most in need; however, there is limited uptake by GPs [26]. Moreover, the dose response observed for the effectiveness of subsidised referrals to allied health care suggest that five visits may not be adequate to facilitate improvement for some individuals [26, 27]. Unlike Australia, the UK and Canada have no out-of-pocket costs for patients seeking allied health services [22].

GPs face many challenges in clinical practice and those working in densely populated areas are particularly at risk of burn-out. This vulnerability has been heightened considering their crucial role in mitigating the impacts of the COVID-19 pandemic within the community [28]. A systematic review evaluating workload in 45 different countries shows that burn-out is common among GPs and importantly influenced by contextual differences within countries [29]. For instance, in primary care GPs are responsible for identifying at risk groups such as refugee women. However, distress and emotional demands often prevail among GPs particularly if healthcare providers find themselves unable to provide the desired patient care due to a lack of resources [30].

To date, there are limited studies exploring GPs perspectives on health care provision for refugee women living with chronic pain [31]. These studies highlight the need for coordinated efforts between providers, policymakers and community organisations due to the complexity of care required. Most studies also propose remuneration be provided to GP’s for the extra time and effort required to manage complex presentations such as chronic pain in refugee patients [32, 33]. A range of strategies for consideration such as improving access to interpreter services, increasing the capacity of the local healthcare workforce, and provide targeted education and support to improve refugees’ health literacy [34–36] were also cited, highlighting the need to explore strategies to improve support systems for GPs in primary care.

Despite the call for enhancing primary care practices, there is a paucity of research that qualitatively explores the perspectives of GPs when managing chronic pain in refugee populations. For this reason, our qualitative study employed a phenomenological approach to explore the lived experiences of GPs providing care for refugee women. Phenomenology is a form of qualitative research that analyses an individual’s lived experience within the world [37]. Adopting a phenomenological approach allows for an in-depth understanding of GPs experiences when providing chronic pain care in refugee-specific contexts. This will help distinguish the enablers and barriers of care within the Australian health care system and contributes to the development of patient-centered approaches that are tailored to the needs of refugee women experiencing chronic pain.

## Methods

### Aim/objective

To explore the experiences of Australian General Practitioners (GPs) managing chronic pain in refugee women.

### Study design

A qualitative methodology using a phenomenological approach was taken to explore GP’s lived experiences managing chronic pain in refugee women [38, 39]. In parallel to GP interviews, qualitative interviews were also conducted with community members from refugee backgrounds who were living with or had lived with chronic pain, to understand their experiences navigating health care in Melbourne, Australia. The findings from this study have been published previously [8].

The reporting of this qualitative study follows the consolidated criteria for reporting qualitative research [40] and was approved by the Monash University Human Research Ethics Committee in February 2022.

### Study participants

Purposive sampling with a snowballing strategy was initially undertaken to recruit GPs who provided primary care services to women from refugee backgrounds living with chronic pain. Eligible participants were accredited with AHPRA and with the Royal Australian College of General Practitioners, who were practicing in Melbourne, Victoria and were required to be conversant in English.

The project was promoted through flyers and information statements emailed to a range of GPs across Melbourne through partner organisations, including emails to clinic practices, newsletters and promotion through each region’s Primary Health Networks website. GPs were invited via email to participate if they had a consultation with at least one patient from a refugee or asylum seeker background. In addition, we invited GPs working specifically in refugee and asylum seeker health to participate. Where appropriate, professional networks of the research team were also contacted for possible recruitment.

### Participant recruitment

Once contact was made with the lead researcher in the research team (AA), the flyer, consent form and plain language statement was sent to the potential participant. Online correspondence allowed for an opportunity to further explain the project and ask questions regarding the information statement. Upon agreement, the interview time and date were decided, and a signed consent form was returned before the interview commenced. Participants were reminded that participation was voluntary and offered $100 honoraria in recognition of the time for study participation.

### Data collection

The study sought to explore experiences of GPs through semi-structured, in-depth interviews (AA). The individual interviews were based on a flexible topic guide (Supplementary file) which was developed by the research team. Question sequencing was flexible, allowing participant responses to guide the course of the interview, while keeping the overall style conversational and situational [41]. The development of the interview guide was based on the research question and guided by our previous research relating to the importance of cultural understanding, trust and compassionate care among refugee women [8]. We oriented the guide to reflect key components of phenomenology and experts in the field such as academics and experienced GPs were consulted in the design. Their insights and knowledge, as well as drawing on the existing work of Harding and colleagues 2017 were instrumental in the formulation of question topics [42]. The interview guide was revised throughout data collection, and informed by iterative data analysis [43]. Interviews concluded once a full and complete understanding of the research topic was achieved and data saturation had been met [44]. Field notes were also used to contextualise the interviews and brought into focus deeper meaning and understanding of the cultural and social context. All transcripts were de-identified before data analysis.

Data were collected between June and November 2022. Interviews were conducted online mostly via Zoom videoconferencing (*n* = 9) or via the telephone (*n* = 1) and lasted between 45 and 90 min.

### Data management

Interviews were audiotaped and detailed field notes were taken. This served as a record of observations, reflections and insights made while conducting interviews to support the interpretation and meaning behind participants responses upon analysis. On completion of interviews, de-identified audio recordings were transcribed verbatim in English, and transferred into a word document. Transcripts were imported into qualitative analysis software NVivo version 12 to help organise the data [45].

### Data analysis

The research team consisted of AA (PhD student with clinical experience in Osteopathy) and three senior qualitative researchers (HB, GR and ES), two of whom are academic family physicians/general practitioners (GPs) (GR and ES). It was important to include GPs in the research team as it offered contextual insights into the health-related topics discussed by participants.

Phenomenology provides the philosophical and theoretical foundation for exploring lived experience, while thematic analysis offers a structured approach to interpret the patterns and meanings of GPs lived experiences [46]. This integrated approach explores qualitative data in a meaningful way and contributes to a more comprehensive understanding of the phenomenon – in this context, physicians’ experiences. The interview data were analysed using the inductive thematic analysis method, with data managed in word and Nvivo [47].

The team met regularly to discuss analyses, and potential categories and sub-category codes. Data was analysed in an iterative manner, using inductive coding early in the analysis process. Codes evolved as the analysis progressed with reflexive interpretations of the data that reflected emerging patterns and themes [48]. Themes based on the pattern of shared meaning were developed, united by a central concept. The themes underwent several revisions by the research team to ensure a correct representation of the concept was being conveyed. Ambiguities were resolved and themes were developed from categories through discussion among the research group members and re-reading of transcripts.

### Reflexivity

Intersecting relationships between participants and researchers play a role in the collection and analysis of qualitative data [49]. Reflexivity refers to the process by which researchers critically examine their own biases and influences to ensure rigour is established in qualitative research [43].

All researchers in this study have prior professional experience working with refugee and/or migrant health issues, therefore, to avoid the influence of any pre-conceived assumptions, subjectivities and/or potential prejudices; self-reflexivity was a particularly important component throughout the research process. Reflexivity during data collection and analysis was acknowledged through reflexive journaling, positionality of the primary researcher (AA), and triangulation for the ongoing and critical examination of the researcher’s influence on the research process. Reflexivity was also achieved more broadly through the regular research team meetings where possible influences and potential biases were discussed. It was also valuable to have the primary researcher (AA) who is a woman and born to migrant parents (born in Lebanon and Turkey), to be involved in data collection and analysis. Experience as a registered Osteopath in clinical practice facilitated a sense of understanding between the primary researcher (AA) and the participants (GPs). This helped to build rapport and created an environment of openness to discuss the intimate details surrounding the many topics of trauma, mental health and their experience of health care provision for chronic pain in populations who are systematically marginalised.

## Results

We interviewed 9 female GPs and 1 male GP. Participants’ time working in general practice ranged from one to 32 years. Clinical context varied: Four worked exclusively in refugee health and the remaining six GPs worked in mainstream general practice or community health clinics across Melbourne. Those who worked exclusively in refugee health had spent a number of years practicing in mainstream or community health clinics before specialising in refugee health. In addition to English, four GPs spoke the languages of some refugee patients. These languages included Nepali, Spanish, French, Cantonese, Mandarin and Hindi (Table 1).

**Table 1:** GP demographic information

Participant	Length of time as GP	Languages spoken	Male/Female	Practice setting
P1	20 years	English	Female	Mainstream practice
P2	2 years	English, Nepali	Female	Mainstream practice
P3	22 years	English	Female	Refugee – specific practice
P4	25 years	English, Spanish, French	Male	Mainstream practice and Community practice
P5	3 years	Mandarin, Cantonese	Female	Mainstream practice
P6	27 years	English	Female	Refugee – specific practice
P7	32 years	English	Female	Refugee – specific practice
P8	25 years	English	Female	Refugee – specific practice
P9	7 years	English	Female	Refugee – specific practice and Community practice
P10	1 year	English, Hindi, Tamil	Female	Mainstream practice

*GP: General Practitioner

We identified three overarching themes from the interview data with GPs. These themes and subthemes are summarised in Table 2. The three overarching themes reflected were: (1) meeting the needs of refugee women with chronic pain; (2) the role of the GP and (3) the challenges of the healthcare system.

**Table 2:** Themes and subthemes

Theme	Subtheme
Meeting the needs of refugee women with chronic pain	Complex presentations
	Building trust
	Listening to people’s story
	Creating culturally safe environments
The role of the GP	Capabilities of clinicians
	Beyond a profession
	Willingness to provide care
Challenges of the healthcare system	Lack of time
	Clinical limitations with Medicare funding
	Navigating the use of interpreters in general practice

*GP: general practitioner

### Theme 1: meeting the needs of refugee women living with chronic pain

#### Complex presentations

The multifaceted nature of chronic pain, combined with the cultural and linguistic barriers often presented with refugee patients, made consultations more complex. GPs spoke of individual expressions of complex illness in refugee women patients presenting with chronic pain and those working in mainstream general practice found this to be particularly challenging to manage:

*“She had just so many complex comorbidities and it was quite overwhelming because of all of these medical problems*,* a lot of her comorbidities were interlinked*,* and centered around chronic pain… I always find those consultations really difficult and really complex to navigate… " P2 female GP*,* mainstream practice*.

GPs also described how patients’ pre- and post-settlement situations also contributed to their pain presentation. Many refugee women often had a history of inadequate or limited access to health care services upon arrival to Australia, adding to the layers of clinical complexity expressed by GPs:

*“It’s really complex*,* chronic pain*,* because women have often had very poor health care*,* very little health care*,* or truncated or fragmented health care when they arrive*,* and many of the things that present with chronic pain actually have other factors that are unique to refugee women” P6 female GP*,* refugee-specific practice*.

Part of the clinical complexity was related to traumatic experiences, which was a frequent and complicating factor in the management of chronic pain. One GP sharing the impact upheaval and uncertainty placed on refugee women’s chronic pain experience:

*“Chronic pain in women is really common*,* particularly in the people who have been living in uncertainty*,* often for very long periods*,* most have been in detention. So the history of trauma in home country and then traumas on the boat trip*,* and in detention and so all that uncertainty often shows up with chronic pain… a lot of the people that we see*,* it’s really clear that the pain is related to trauma” P3 female GP*,* refugee-specific practice*.

Moreover, language barriers in patients who often presented with pain as a consequence of trauma made clinical care even more challenging as one GP explained:

*“[They are] actually being expected to talk about traumative experiences and these complex complaints of pain and other stuff that your concerned about and in a language that is your second*,* third*,* fourth or fifth language” P3 female GP*,* refugee-specific practice*.

#### Building trust

Establishing rapport and trust with refugee women patients was important for GPs as it formed the foundation for a therapeutic relationship that was grounded in empathy and understanding. GPs placed considerable value on the doctor-patient relationship and believed it significantly impacted the patient’s ability to communicate concerns and consequently the quality of medical care provided:

*“The first is to establish a good therapeutic relationship*,* and that takes time*,* that takes a lot more time than actually doing the medical stuff… it may take a few consultations to really understand the patient*,* their life*,* how their condition impacts on their life*,* and I think if you are able to do that well*,* the patient will engage with you*,* and come back and trust you” P2 female GP*,* mainstream practice*.

However, building trust was challenging as GPs observed that the narratives of many patients revealed a history in which figures of authority had perpetrated harm, leading to many patients exhibiting a sense of distrust:

*“I mean there’s a large cultural component*,* so often people who come from countries where people in positions of authority have done bad things to them*,* there’s that distrust” P3 female GP*,* refugee-specific practice*.

Trust played an equally important role in enabling GPs to appropriately manage chronic pain. Chronic pain management often involves multiple factors and requires ongoing care between patients and often several health care providers. Trust helped GPs tailor their care, educate refugee women patients about their condition and led to a more comprehensive chronic pain management plan:

*“You have to rebuild the trust again… once they’ve had a bad experience*,* don’t feel like they’ve been heard or if their pain continues*,* and explain to them that it’s an involved cause*,* it’s not purely a physical cause*,* I think that sometimes it’s difficult for them to understand that we can’t just give a tablet and make the pain go away” P7 female GP*,* refugee-specific practice*.

Contrastingly, some GPs also spoke of situations where patients showed unwavering trust towards doctors’ medical decisions. Whilst this perception was not necessarily preferred, it was in contrast to mainstream Australia where the doctor-patient dynamic often resembles a consumer-provider relationship.

*“They tend to have a very paternalistic view of medicine*,* that the doctor’s always right*,* the doctor knows best. In mainstream Australia it’s a very horizontal relationship*,* it’s like a consumer*,* you go with an ailment*,* you get the treatment you want and if you don’t you just move onto another doctor seeking the same treatment.” P10*,* female GP*,* mainstream practice*.

#### Listening to people’s story

GPs acknowledged that building rapport and establishing trust was important. Active listening was an important strategy to achieving a therapeutic relationship and many participants highlighted that their approach to caring for their patients who presented with complex clinical needs such as chronic pain was grounded in the art of listening:

*“I’m really focused on hearing people’s stories*,* it’s so easy to make assumptions for what’s happening for a person based on physical symptoms*,* but I try and carve out time during an initial consultation to say*,* ‘tell me about what life is like for you*,* what was life like for you before you came here?’. To hear people’s stories*,* I think that’s incredibly powerful. One*,* because people feel heard*,* and two because you understand the context of what has been going on for that person.” P9 female GP*,* community practice*.

Many refugee women patients had a complex history of trauma or protracted unrest, for this reason GPs highlighted that it was especially important to listen to refugee women’s stories relating to pain, as both trauma and pain were often interlinked.

*“You’re there to listen to their story and understand what might be just back pain for one patient… could be completely debilitating for the other patient because it has to do with pain perception… affected by life experience… you need to give them time to tell their story and understand their story you know even if it’s completely or [seemingly] unrelated.” P10 female GP*,* mainstream practice*.

#### Creating culturally safe environments

As a result of the complexities arising in GPs consultations with refugee women exhibiting chronic pain, almost all GPs working specifically in refugee health emphasised the importance of establishing culturally welcoming environments:

*“[I] recognise that there are things that I may not know or understand about their language or their culture and try to be open to learn from them too… I do try and build rapport also try and let them know that this is their space and that what we talk about stays in that space and it’s private” P8 female GP*,* refugee-specific practice*.

Some GPs also spoke of the importance of paying attention to non-verbal communication through noticing and attending to body language. Attending to non-verbal cues helped doctors connect with their patients and helped inform culturally appropriate ways to educate patients. Non-verbal cues such as body language were also employed by doctors themselves to enhance their care delivery:

*“Consultations [are] a bit more challenging because often I will have to use more body language to convey my care and develop rapport… sometimes I have to be very slow in my speech*,* use pamphlets and take extra time*,* so longer appointment times*,* and see them a few times before I fully comprehend what their problem is” P5 female GP*,* mainstream practice*.

In some instances, cultural concordance helped doctors establish a stronger connection with their patients. It enabled doctors to align their cultural knowledge, beliefs, and practices with those of their patients. According to one GP, sharing a cultural background helped them to understand the socio-cultural influences that can impact patients:

*“Because I’m also of a CALD [culturally and linguistically diverse] background*,* I can feel like I can relate to them in a sense whereby*,* potentially sociocultural things trump or predominate their life over things like their job*,* or going to the doctor " P2 female GP*,* mainstream practice*.

Nevertheless, doctors who shared a cultural background with their patients’ also described difficulties when it came to maintaining professional boundaries:

*“When I am seeing for example patients [from the same culture]*,* I find it really difficult to maintain… boundaries… being their GP versus being a person of the community is really difficult” P2 female GP*,* mainstream practice*.

### Theme 2: the role of the GP

#### Capabilities of clinicians

Many GPs felt unprepared when it came to the unique challenges specifically regarding refugee women who exhibited chronic pain. GPs spoke of needing more refugee specific education in both undergraduate general practice training and government-level policies that prioritise and support educational initiatives:

*“There isn’t a lot of content in this area*,* it wasn’t in GP training there was nothing specific about caring for refugee people*,* or how to deliver the care for these women” P5 female GP*,* mainstream practice*.

Some GPs also highlighted that their skills working in refugee health was aided by being a member of the migrant community themselves. This helped to contextualize concepts of cultural safety and competency learned during their GP training:

*“It’s based a lot on personal experience and being a part of a migrant community… honing in on the skills we were taught in our training that relates to approaching culturally diverse people… even our Aboriginal and Torres Strait Islander health training has been useful to contextualize what cultural safety and sensitivity and competency looks like*,* and you can apply that to any one [or] culture” P2 female GP*,* mainstream practice*.

Moreover, many working in both mainstream and refugee specific health centres spoke of informal learning channels such as colleagues and experiential observations to supplement their understanding and expertise around care provision for refugee women presenting with chronic pain:

*“[From] experience and a lot of informal education from mentors and colleagues*,* a lot of looking stuff up as I went along*,* looking for appropriate guidelines or reading… I honestly felt incredibly unsupported when I started doing this work and had to kind of find people and resources to educate myself around it” P9 female GP*,* community practice*.

Contrastingly, irrespective of the setting in which they practiced (i.e., specific refugee health, mainstream general practice or community health) all GPs felt competent managing the clinical condition of chronic pain. One GP provided a routine example of how multidisciplinary care would be enacted when chronic pain was presented in practice:

*“What I’ve done for most people as a baseline foundation is coordinate their care between a multidisciplinary environment*,* that’s the only way you can really manage chronic pain well… it’s really about referring yes to allied health*,* and then linking them in with a pain specialist*,* mainly for de-prescribing… then I refer them onto a psychologist to manage the comorbid mood disorder*,* so that’s where I start generally*,* and then the flow on effects of that depend on the individual circumstance*,* so depending on how that effects their life then I’ll think about what else they need.” P2 female GP*,* mainstream practice*.

#### Beyond a profession

GPs working in refugee health shared accounts of cautiously and selectively bending professional boundaries for patients who had experiences of pre-migration trauma. One doctor giving the example where they gave personal contact information to a patient who faced isolation, loneliness and needed additional support.

“*For this [one] woman*,* because it was Christmas and everything was closed*,* I gave her my mobile number and email address*,* and I don’t do that all the time*,* because I’m very conscious of boundaries but there are some people and when they are so isolated*,* and alone and just need to be able to contact you*,* then I do actually bend the rules… with some patients*,* particularly with people who have been through really traumatic experiences and need to feel safe” P3 female GP*,* refugee-specific practice*.

Another GP described an unconventional approach of learning words in their patient’s native language, to foster a sense of connection and comfort:

*“I’ll say my one word of Arabic ‘Insha’Allah’ and they will laugh hysterically*,* so showing some interest or knowledge of culture and how that might impact on their medical health decision making and trying to learn a few words of the language” P6 female GP*,* refugee-specific practice*.

Despite ethical challenges, some GPs extended beyond the boundaries of professionalism to ensure patients received appropriate care. One example of this commitment is the reality of working beyond standard billing hours to attend to the needs of refugee patients.

*“I think I spent about two hours with the health and bicultural worker*,* to get him seen in hospital*,* talking to various doctors to work out the best way to get him into hospital and get him seen quickly…and for that two hours for both mine and the bicultural workers time we got paid 39$” P9 female GP*,* refugee-specific practice*.

In order to deliver appropriate care and maintain professional boundaries, many GPs who served patients from disadvantaged backgrounds either worked pro-bono or in community health clinics where they were inadequately compensated for their time. Although this was common in refugee health care, GPs were also aware that this system was not representative of the care delivery possible by all GPs across Australia:

*“The way that we work is certainly not representative of what’s possible for every other GP in Australia*,* I mean the cost is we don’t get paid… we do it voluntarily*,* but the benefit is that you do actually get to deliver really good quality care*,* so there is a really huge gap there” P3 female GP*,* refugee-specific practice*.

#### Willingness to provide care

There was a shared sense of purpose reflected among GPs who worked specifically in refugee health that extended beyond professional responsibility:

*“[it] is worthy work… I feel like at the end of the day I need to be proud of myself*,* that I have made a difference*,* even if it’s a small difference*,* even if it’s something like relating to the patients pain*,* it’s not just by prescribing medicine*,* it’s by understanding their story*,* showing that you genuinely care*,* and just making a small difference to that patients life.” P10 female GP*,* mainstream practice*.

However, the multifaceted nature of chronic pain management when combined with the contextual challenges of refugee women patients, impacted some GPs willingness to provide care.

*“From my experience*,* people from culturally diverse backgrounds*,* especially women*,* do present a bit later*,* and they don’t have the full understanding of the value of medicine and the acuity and complexity of it” P5 female GP*,* mainstream practice*.

Those working in refugee specific practices highlighted how complex chronic pain was better managed with clinical supports such as refugee health nurses:

*“I can speak to one of our refugee health nurses and say ‘can you find a physio for this patient can you organise this x-ray*,* can you ring them in a week’s time*,* and see how they’re going with their medications*,* can they come back in*,* or can you follow them up and can you make an appointment for them to see me in a month’s time’ and then you feel like your encasing that person in a bit more support” P7 female GP*,* refugee-specific practice*.

Given the many clinical challenges presented, some doctors described the ‘heart-sink’ phenomena in reference to refugee women presenting with chronic pain. This term has been used in medical discourse to describe how doctors can have a feeling of helplessness in the face of complex patients where resolution or healing is unlikely [50]:

*“It’s clear that certain GPs won’t see certain conditions… certain people are more adept to treat certain conditions than others*,* so doctors who have a heart-sink patient*,* an abysmal expression*,* that patient that when you see them*,* your heart sinks because it’s going to be such a challenging consultation*,* it’s an appalling concept but it’s well entrenched in doctors psyches. I think that where the patient is seen as ‘heart-sink’ than the doctors approach needs to change because it’s not correct… " P4 male GP*,* community practice*.

As a result, some GPs expressed burnout as a consequence of the frustration arising from the challenges arising in clinical practice:

*“Being a GP is actually really tough work and a lot of people get burnt out as a consequence*,* cause we’re expected to know a little thing about a lot of stuff*,* [many] might only have a few patients in their clinic who are of refugee background so people don’t delve deeper because they’re so busy and overwhelmed with their workload…” P3 female GP*,* refugee-specific practice*.

### Theme 3: challenges of the health care system

#### Lack of time

Many GPs believed that their willingness to provide care was centered around having sufficient time to provide comprehensive care to refugee women experiencing chronic pain.

*“I think for GPs it can be challenging to make the time for patient education*,* make the time for calling an interpreter… practising good medicine takes time. Unfortunately*,* experienced GPs are so time poor and they have so many complex patients*,* they would be running two hours behind and everyone would be unhappy” P10 female GP*,* mainstream practice*.

Almost all GPs believed that time was a clear barrier to effective chronic pain management in refugee populations. GPs spoke of needing more time particularly because chronic pain in women from refugee backgrounds also presented with a complex history of trauma and overlapping health issues:

*“What would help me provide care for these women*,* I would say is definitely time*,* I would prefer to have more time to speak*,* so time is a big factor” P5 female GP*,* mainstream practice*.

Time was also a complicating factor in the management and coordination of chronic pain. Many GPs highlighted concerns around the long wait times and restrictions placed on pain clinics which further challenged clinical care:

*“Pain clinics are getting more restrictive and harder to access*,* and more… sort of yes restrictive in what they will accept and also longer waiting lists” P4 male GP*,* community practice*.

#### Clinical limitations with medicare funding

Most doctors working in refugee health, or in community health settings felt that the lack of funding in mainstream GP clinics restricted scope of care. GPs spoke of challenges with the current health care system as it incentivised quick care which was often in contrast to the care required to manage chronic pain in refugee women:

*“Medicare funding has been really stagnant for a very long time*,* it funds quick care…a six minute medical consult is inadequate for most things*,* but for someone who can’t advocate for themselves*,* can’t spit out a medical history really quickly*,* tell the doctor what they’re needing and wanting and worried about*,* which is almost all of my patients… it takes time and it takes tact and it takes kind of cultural contextualization. That system doesn’t work for patients who are refugees*,* who are women trying to seek care for complex conditions " P9 female GP*,* community practice*.

Similarly, GPs spoke of general practice moving towards “churn and burn” medicine which does not supports doctors’ needs. This speaks to a growing trend seen in general practice where GPs are required to see a high volume of patients within limited timeframes, and without sufficient resources:

*“The [clinical] systems weren’t supportive of GPs doing the work*,* there was this pressure from management to see more and more patients*,* which is that kind of churn and burn type medicine*,* it’s really antithetical to looking after someone who has complex medical and health needs but also might have complex communication needs” P9 female GP*,* community practice*.

Medicare also offers CDM plans to support GPs coordination of chronic diseases such as pain. CDM plans assists GPs management of chronic pain by providing patients with five annual visits to an allied health professional at a subsidised cost. However, despite the perceived benefits, GPs stance on CDM plans were mixed. Many felt that funding was inadequate, with little actual benefit for the patient:

*“I mean their role is to get five*,* rather badly subsidised sessions of allied health [only $55 dollars per session of allied health reimbursed]… for too many [patients]*,* it doesn’t make them affordable at all ” P4 male GP*,* community practice*.

In contrast, some GPs explained that the benefits of CDM when used to extend appointment times allowed consultations to be better funded. However, this was in part dependent on whether GPs were well supported by administrative staff that could facilitate the processes of CDM plans, as one doctor explained:

*“Medicare doesn’t delineate for where people are from or what their background is*,* …[incorporating] a chronic disease management plan as a part of the process means then some of the following consultations are more well-funded… but those take time in themselves to do*,* but it’s a matter of having those systems in place at your clinic…” P1 female GP*,* mainstream practice*.

As a result, many GPs described not feel adequately supported by the health care system when managing refugee patients presenting with complex health issues such as chronic pain:

*“That system doesn’t work for patients who are refugees*,* who are women trying to seek care for complex conditions” P9 female GP*,* community practice*.

#### Navigating the use of interpreters in general practice

Navigating interpreter use in general practice varied. Whilst all GPs described professional interpreter use as an integral component to culturally appropriate care, experiences with the actual use of interpreters in the real world of general practice were varied. Some GPs described the use of interpreters as a simple and straightforward:

*“I don’t think it’s difficult to provide care for someone from culturally diverse communities*,* I mean we’re doctors that’s our job*,* and finding interpreters are not difficult*,* 13 14 50*,* call up and you get an interpreter*,* it’s super easy” P10 female GP*,* mainstream practice*.

However, many other GPs working in mainstream GP clinics described the process as burdensome as one GP shared:

*“It’s challenging*,* it takes so long*,* way more that 2 minutes and often you have to set it up in advance… if you have good reception staff or good administrative staff it will happen very efficiently. You spend the majority of your consult trying to get a hold of the interpreter*,* then you have them on speaker and you’re also doing an examination*,* and then explaining the diagnosis and everything… it’s really difficult” P2 female GP*,* mainstream practice*.

The adoption of interpreter by GPs was heavily dependent on the presence of well-established clinical support systems. An example provided by a GP illustrated that interpreter use when coordinated by administrative staff, could alleviate many clinical challenges that present with refugee women who exhibit chronic pain:

“*If for example reception*,* the administrative side of the clinic*,* if they’re more willing to support me*,* like with the interpreting service to help with these women that would encourage me more to see them” P5 female GP*,* mainstream practice*.

Doctors less commonly relied on family for interpreting. Whilst all GPs highlighted the harms that may result from having family and friends interpret on behalf of their patients, others felt that in certain situations and with the time-constraints often imposed on GPs, family members were better than no interpreting service at all:

*“[Family] are less difficult to navigate than the interpreting service to be honest but I’m not sure that the accuracy is on par… it’s really hard… [you have to] weigh up the risk and the benefit of not being examined and if we delay this and it affects their health*,* so it’s really that risk-benefit kind of conversation” P2 female GP*,* mainstream practice*.

## Discussion

Our phenomenological study explored GPs lived experience providing care for refugee women living with chronic pain. Whilst GPs welcomed care, they were challenged by the complex needs of refugee women, the role of the GP profession and the hurdles associated with the Australian healthcare system. The experiences of GPs suggest that there are numerous difficulties that arise when providing care for refugee women patients presenting with chronic pain relating to culture, language, inadequate administrative resources and additional system level hurdles such as time and funding. As research suggests, it became evident that establishing trust and finding meaning in their work held significant importance for doctors [51]. Notwithstanding the challenges of chronic pain management, GPs found it difficult to abstract chronic pain from the unique characteristics of refugee women and the numerous complexities that accompany a fragmented life. Many women from refugee backgrounds arrive in host countries such as Australia following periods of considerable unrest, disruption and emotional turmoil. Thus, GPs found it difficult to address chronic pain in refugee women patients without firstly taking into account the broader contextual challenges associated with resettlement and displacement [8]. These complexities faced by GPs highlight an urgent need for improved clinical supports and a more comprehensive approach to health care provision for women of refugee background seeking care for chronic pain.

Central to providing compassionate, ongoing and equitable care to refugee women is the concept of cultural safety to ensure refugee women patients are able to meaningfully engage in medical dialogue. Patel’s study on appropriate care delivery for refugee and asylum seekers in Australian primary care highlights the importance of drawing on available resources [52]. Resources such as shared language and establishing continuity through follow up appointments help to build trust over time [53]. Petrocchi and colleagues (2019) also suggest that medical trust is positively associated with adherence to treatment, continuity of care and ultimately better patient outcomes [20, 54, 55]. However, building a therapeutic relationship that was grounded in trust required time, and the scarcity of time in general practice was frequently cited as a major obstacle to effective clinical care [56].

Tsiga and colleagues (2013) study on workplace stress demonstrate how time can influence GPs’ capacity to adhere to clinical care standards such as following medical guidelines, comprehensive history assessments, and patient education [57]. Furthermore, other organisational factors that shape routine primary care can impact health outcomes for patients and doctors alike. Cultural factors associated with refugee patients such as limited health literacy, language discordance and the need to explain system-related information led to increased consultation times and financial losses for GPs and their practices. However, there are several clinical support systems that help to alleviate time pressure exhibited in primary care. In line with our findings, Davison and colleagues (2023) suggest that clinical supports such as refugee health nurses who assist with intake procedures, coordinating chronic disease management plans, mental health care plans and interpreter use, allow doctors more time to attend to their patients’ more complex health needs [58]. This may also help mitigate economic challenges associated with managing complex conditions in underserved communities and incentivise other GP clinics to accept more patients from refugee backgrounds.

Trauma and resulting psychological conditions such as Post Traumatic Stress Disorder (PTSD) was a frequent and complicating factor in the management of chronic pain [59]. In line with our findings, both Miro and colleagues (2008) and Shaw and colleagues (2010) posit that PTSD may potentially contribute to a heightened pain perception and pose as a risk factor for the onset of chronic pain [60, 61]. To effectively address the complex presentations and meet the unique needs of refugee women exhibiting chronic pain, it was essential for GPs to engender trust and listen to their patients’ stories. Whilst many GPs did not report implementing trauma informed care, many of the practices and values being applied by participants in their efforts to deliver appropriate care to refugee women patients were consistent with the principles of trauma informed care. Trauma informed care acknowledges the need to understand a patient’s life experiences and how the experience of trauma impacts someone and how they engage with health care services [62]. The values of trauma informed care, as suggested by Bowen and Colleagues (2016), include; safety, trustworthiness, transparency, collaboration and peer support, empowerment and choice [63]. Brook’s study of trauma informed care in general practice within a women’s health center suggest that recognising the impact of trauma in consultations offers long-term and safe relationships among patients and doctors [64]. Furthermore, it highlights a holistic model of care to manage the health consequences of adversity and trauma [64]. Doctors’ ability to embrace a person-centered approach that recognises the significance of trauma informed care within clinical practice has enormous potential for improving refugee women’s chronic pain outcomes in general practice.

Although the importance of medical interpreters was acknowledged by all GPs, those working in mainstream practices recounted numerous challenges when integrating phone-based interpreting services during routine care with refugee women patients. According to The Royal Australian College of General Practice guidelines, qualified medical interpreters should be the interpretation medium of choice and the College advises caution when using family or friends to interpret [65]. In line with our findings, White and colleagues (2018) highlight that language discordant clinical encounters can seriously compromise patients’ quality of care and health outcomes, particularly when managing complex medical conditions such as chronic pain [11, 66]. Contrastingly, an Australian study exploring the experiences of refugee women seeking care for chronic pain showed that a number of participants expressed a greater sense of comfort when relying on family members rather that professional interpreters for language interpretation [8]. Similarly, national GP data shows that one in five GPs continue to used family or friends to interpret during consultations in a language other than English [67]. Reasons underlying GPs preferences for informal interpreter use over professional healthcare interpreters vary; however, potential reasons include the availability of healthcare interpreters, the necessity of scheduling in advance and time constraints faced by doctors [68–74]. Other contributing factors include patient’s privacy concerns, familiarity with family members, confidence derived from one owns language proficiency, challenges accurately assessing the need for an interpreter and a lack of familiarity with the interpreter service or booking system [69–72]. Notwithstanding these limitations, many GPs in our study underscored the importance of a neutral information bridge between doctors and patients particularly when managing chronic pain.

According to the inverse care law, the availability of high-quality medical care is inversely related with demand for it within a given population [75, 76]. The reasons behind this disparity in quality healthcare received by individuals from disadvantaged backgrounds, comparted to their wealthier and healthier compatriots is complex; however, supply-side factors contribute to this phenomenon [77, 78]. Supply side influences such as resource allocation, healthcare infrastructure, workforce distribution and funding mechanisms tend to favour more affluent regions and create challenges for individuals such as those from refugee backgrounds to receive the same high quality medical care. GP participants that worked in environments that were aligned with refugee women’s needs found that the clinical demands when working with refugee women presenting with chronic pain were easier. Moreover, many felt that their work helped to minimise health disparities by advocating for equitable health care access among individuals who faced systematic disadvantages. However, this vocational commitment by some GPs who worked exclusively with disadvantaged groups like refugee women came at a cost. Oftentimes, GPs who worked pro bono or in community health felt they were insufficiently remunerated.

for the time they spent with their patients [12]. A review by Goupil and Colleagues (2020) examined the effectiveness of pro bono health care delivery in addressing the critical gap in healthcare services. Their findings indicate that pro bono initiatives have shown considerable success in addressing the gap created by limited access to primary health care for underserved populations [79]. Nonetheless, the need to work with inappropriate remunerated limits the number of GPs who can engage in refugee work. For instance, those who have alternative sources of income, such as a partner, may be more likely to afford this limitation. The current reliance on altruism, or selfless dedication by GPs to sustain the system may not be an effective strategy in the long-term [80–82]. Thus, it becomes increasingly challenging for GPs who lack additional financial support to commit fully to their professional duties and reinforces broader implications for the primary care workforce.

General practice inherently presents stressors that subject many GPs to high levels of stress, resulting in adverse consequences such as frustration and burnout [83, 84]. The effects of burnout on individuals’ overall well-being are substantial; however, among doctors, its significance is far greater due to its potential to detrimentally affect patient care. Riley and Colleagues (2017) highlight how stress and uncertainty at work can contribute to distress and dysfunction among adults. GPs who are under-resourced are not able to offer the same level of patient-centred care for refugee women patients presenting with complex issues such as pain. The increase in workloads and demands on GPs demonstrates a need to re-evaluate policies and agendas affecting primary care both in GP training and within the public health system. As Linzer and colleagues (2015) suggest, a supportive work environment supplemented with educational opportunities and system level interventions that help to mitigate clinical constraints can improve doctor’s resilience and confidence within the workplace [85]. This is supported by Shanafelt’s research who suggests that enhancing meaning in work may also serve as a prescription for mitigating physician burn out and better patient centered care [86].

There are some limitations of this study that should be noted when considering the findings. The study was conducted between June 2022 and November 2022, under a high stress period for GPs inundated with COVID-19 cases. This meant that under time and feasibility constraints, the interviews were conducted virtually which may have created some limitations around disclosure and open dialogue for some GPs. A further limitation arises from the fact that networks used to recruit participants and snowballing sampling may have biased our findings as many may share similar professional backgrounds, experiences and perspectives, restricting the breadth of insights captured in the study. Our sample size was limited meaning it may not accurately mirror the experiences of all GPs or the generalisability of our findings to a broader context. Furthermore, the specific challenges experienced by GPs may not be universally applicable to other healthcare professionals with different roles, specialities or interests. Notwithstanding these limitations, the depth and richness of data gained from the interviews produced valuable and meaningful insights of the GP experience. Lastly, to ensure diverse perspectives and experiences were included in our sample, the research team regularly engaged in reflexivity to critically examine our own professional assumptions and biases.

## Conclusions

Understanding GP experiences offers valuable insights that can inform targeted interventions and policies to enhance the quality of care for refugee women living with chronic pain. Future policies should look to enhance collaboration between GPs and refugee community members and liaise with refugee specific health professionals to better care for refugee women patients in mainstream primary care. These initiatives should prioritise administrative support systems such as refugee health nurses and work towards streamlining the integration of medical interpreters in general practice. Furthermore, improved awareness and uptake of existing Medicare fundings streams such as CDM, mental health and refugee health assessments plans are needed to effectively address the distinct requirements of refugee communities. Lastly, postgraduate curricula should look to integrate more refugee health topics to better equip GPs with the necessary skills and knowledge to bolster their confidence and clinical acumen when providing care for refugee women facing complex chronic pain.

## Electronic supplementary material

Below is the link to the electronic supplementary material.

Supplementary Material 1

## Data Availability

Transcripts from the interviews are confidential and not publicly available. A subset of de-identified data is available from the corresponding author.

## Abbreviations

GP: General practitioner
PTSD: Post Traumatic Stress Disorder
