# Supplementary material for: Doctors’ experience providing primary care for refugee women living with chronic pain: a qualitative study
Source: BMC Health Serv Res. 2024 Sep 27;24:1117. doi: 10.1186/s12913-024-11506-x (PMC11429581; doi:10.1186/s12913-024-11506-x)
Supplement: Supplementary file 1 — Supplementary Material 1 [file 12913_2024_11506_MOESM1_ESM.docx]

# Interview Guide

1. Can you tell me a bit about your experiences with women from culturally diverse background, particularly those from refugee backgrounds?
   - We are particularly interested in your experience with culturally diverse women who are living with chronic pain, what has been your experience with this?
   - Do you have a patient or story in mind you could share?
2. In your opinion, how easy is it for Culturally diverse women to get primary care?

Follow-up

- What do you think there are barriers for women?
- What about cost? What about cost for further care for pain services?
- What do you think *your practice* does to support culturally diverse women living with chronic pain?
- Are there any the strategies you provide in your care to prevent chronicity?

– what are the next steps i.e. Facilitation of access to specialty pain clinics/osteo/physio etc

1. From our previous findings, many of the refugee women we spoke to mentioned that feeling comfortable around their GP was very important. What would you hope that they could receive when seeing you?

Follow-up

- Many women in our study also mentioned being followed-up is an important aspect of their care, tell us about how you follow up with your patients?
- What do you think you bring to the care of culturally diverse women who have chronic pain?

## What makes it harder to care for these patients belonging to culturally diverse communities?

1. Thinking a bit about the structure of the clinic itself and the potential needs of culturally diverse women, what are the ways in which your clinic accommodates?

- Has telehealth influenced the ways in which culturally diverse patients access care at the practice?

1. What about the issue of language, how do you manage language barriers?

Follow-up question

- - Does your clinic offer interpreters?
  - Are you able to book in-person translators for your appointments? - is this something that you think could be useful?

1. Thinking about contextual elements such as gender in refugees and culturally diverse people, how do you deal with this from the perspective of the provider?
2. What are some of the things that would make it easier for you to manage chronic pain in refugee women in general practice?
   - What about Chronic Disease Management plans or Mental Healthcare Plans?
   - Much of our previous interviews both with GPs and consumers suggested that it was difficult to engage CALD and refugee women in their own health care due to competing priorities. What has been your experience with this?
3. What do you think are the factors that influence a GP/practice’s capacity or willingness to provide care to patients culturally diverse women experiencing chronic pain?
4. What do you see as the priorities for GPs who treat women belonging to culturally diverse communities and who are living with chronic pain?

Follow-up questions:

- Is there a need to improve the GP service for culturally women experiencing with chronic pain?
  - Many of the women in our study mentioned that feeling heard was an important aspect of trust in their relationship with their GP. What have your experiences been with this?

## Demographics

1. Now I would like to ask a few questions about yourself. How long have you been working in this practice?
   - How long have you worked as a GP?
   - Do you speak languages other than English?

- Do you sometimes speak <<this language>> with your patients? Why?
  - Where does your current knowledge and skills in caring for patients with culturally diverse women come from?
  - Have you done any specific training in the past that helps you working with vulnerable populations?
  - Can you give me an example of how this training helped you dealing with culturally diverse patients?
